# Usefulness of Cathepsin S to Predict Risk for Obstructive Sleep Apnea among Patients with Type 2 Diabetes

**DOI:** 10.1155/2020/8819134

**Published:** 2020-09-25

**Authors:** Wanwan Wen, Haili Sun, Yunxiao Yang, Yifan Jia, Fang Fang, Yanwen Qin, Ming Zhang, Yongxiang Wei

**Affiliations:** ^1^Department of Cardiology, Beijing Anzhen Hospital, Capital Medical University, Beijing 100029, China; ^2^Department of Otolaryngology Head & Neck Surgery, Key Laboratory of Upper Airway Dysfunction-Related Cardiovascular Diseases, Beijing Institute of Heart, Lung and Blood Vessel Diseases, Beijing Anzhen Hospital, Capital Medical University, Beijing 100029, China; ^3^Beijing Institute of Heart, Lung and Blood Vessel Diseases, Beijing Anzhen Hospital, Capital Medical University, Beijing 100029, China

## Abstract

**Background:**

Obstructive sleep apnea (OSA) was highly prevalent in patients with type 2 diabetes (T2D). Cathepsin S (CTSS), a cysteine protease, is involved in the inflammatory activity in T2D and hypoxia conditions. The aim of the study was to evaluate whether CTSS could be involved in the inflammatory reaction of OSA in patients with T2D.

**Methods:**

We included 158 participants in this study matched for age, gender, and body mass index in 4 groups (control, non-OSA&T2D, OSA&non-T2D, and OSA&T2D). After overnight polysomnography, we collected the clinical data including anthropometrical characteristics, blood pressure, and fasting blood samples in the morning. Plasma CTSS concentration was evaluated using the human Magnetic Luminex Assay.

**Results:**

Compared with the control group, both the non-OSA&T2D group and the OSA&non-T2D group showed higher CTSS levels. Plasma CTSS expression was significantly increased in subjects with OSA&T2D compared to subjects with non-OSA&T2D. The OSA&T2D group had higher CTSS levels than the OSA&non-T2D group, but there were no statistically significant differences. Plasma CTSS levels showed significant correlation with the apnea-hypopnea index (AHI) (*r* = 0.559, *P* < 0.001) and plasma fasting blood glucose (*r* = 0.427, *P* < 0.001). After adjusting confounding factors, plasma CTSS levels were independently associated with the AHI (Beta: 0.386, 95% confidence intervals (CI): 21.988 to 57.781; *P* < 0.001). Furthermore, we confirmed the higher pinpoint accuracy of plasma CTSS in the diagnosis of OSA (area under the curve: 0.868).

**Conclusions:**

Plasma CTSS expression was significantly elevated in the OSA&T2D group and was independently associated with the AHI; it could be a biomarker with a positive diagnostic value on diagnosing OSA among patients with T2D.

## 1. Introduction

Obstructive sleep apnea (OSA) is a common disease that is characterized by repetitive collapse of the upper airway during sleep resulting in snoring, sleep disruption, nocturnal chronic intermittent hypoxia (CIH), and oxidative stress, which cause endothelial dysfunction and inflammatory reaction [1–3]. Previous studies have reported that OSA severity is being gradually recognized as a possible cause of glucose intolerance, insulin resistance, and diabetes [4, 5]. Moreover, it has also been reported that diabetes could increase the predisposition for OSA [6, 7]. Furthermore, multiple epidemiological studies showed that around 50%-90% of patients with type 2 diabetes (T2D) have OSA; thus, screening for OSA could be crucial for patients with T2D [8–10]. The American Academy of Sleep Medicine (AASM) clinical practice guidelines suggest that screening questionnaires and facility-based polysomnography (PSG) can be used to screen and diagnose OSA in adult patients with suspected OSA [11]. However, due to the lower specificity and accuracy, screening questionnaires can just be used to preliminary screen OSA for suspected sleep disorder. Moreover, the usage of PSG was comparatively expensive and time-wasting. The PSG was also impractical for the clinical evaluation of large at-risk populations [12]. Given the above factors, comprehensive analysis of the molecular mechanism under OSA conditions is so paramount, which contributes to the detection and diagnosis of this sleep disorder in patients with T2D.

Cathepsin S (CTSS), a cysteine protease, participated in antigen presentation and cellular proteolysis [13, 14]. Moreover, prior studies have proposed a causal relationship between CTSS activity and inflammatory reaction; higher circulating levels of CTSS are associated with increased inflammatory activity [15, 16]. Experimental studies suggest that CTSS activity is involved in prediabetic alterations in the liver and autoimmune diabetes [17, 18]. Moreover, CTSS activity has been implicated to play an active role in inflammatory reactions and is upregulated in hypoxic conditions [19, 20].

Based on previous experimental reports suggesting a role for CTSS activity in the inflammatory activity in T2D and hypoxic conditions, we hypothesized that plasma levels of CTSS would be a biomarker for diagnosing OSA in patients with T2D. Accordingly, we investigated the association between plasma levels of CTSS and the occurrence and severity of OSA in a hospital-based population. As a second step, we explored the association between CTSS and OSA in patients with T2D.

## 2. Materials and Methods

### 2.1. Study Design and Population

The observational cross-sectional study was performed in Beijing Anzhen Hospital. The study protocol was supported by the Medicine Ethics Committee of Beijing Anzhen Hospital and complied with the Declaration of Helsinki. This study was registered with the Chinese Clinical Trial Registry (No. ChiCTRROC-17011027). In total, 180 participants were recruited and underwent overnight full PSG in the Sleep Laboratory between March 2017 and November 2018. Patients with respiratory disease, type 1 diabetes, gestational diabetes, coronary heart disease, hypertension, hyperlipidemia, active hepatitis, cancer, and drug or alcohol addiction were excluded from this study, or other known major diseases were precluded from the study [21]. Participants who were younger than 18 years, refused to sign the consent form, or have incomplete information were also excluded. Finally, we selected a total of 158 eligible participants. Data on participants' baseline demographics were collected from the questionnaires. Anthropometrical characteristics included weight (kilograms), height (meters), body mass index (BMI) (kg/m^2^), blood pressure, and left ventricular ejection fraction which were calculated by standardized instructions. Current smokers were defined as participants who were smoking or stopped smoking for less than one year before enrollment in this study. Drinkers were participants who consumed alcohol more than three days a week. Serum lipid, high-sensitivity C-reactive protein (hs-CRP), and other indicators were measured as previously described [22, 23].

### 2.2. OSA Assessment

Each eligible subject underwent a comprehensive sleep assessment. Neck circumference (cm) was measured between the midcervical vertebra and midanterior neck by a flexible tape [24]. The Epworth Sleepiness Scale (ESS) was used to assess the risk of daytime sleepiness, which was measured for each study subject [25]. All participants underwent overnight PSG (SOMNOscreen; SOMNOmedics GmbH, Germany) supported by the US Food and Drug Administration. Alcohol or sleeping medicines must be strictly prohibited before the PSG examination. The SOMNOscreen device continuously monitored and recorded abdominal and chest motion, respiratory effort airflow, arterial oxygen saturation, electroencephalogram, and electrocardiogram through computerized polysomnograms. Based on the 2012 standards of the AASM criteria, sleep stage and cardiopulmonary events were manually evaluated by certified technicians [26]. The definition of hypopnea was greater than 3% oxygen desaturation and sustaining for at least 10 s. The definition of apnea was airflow complete cessation or a 90% airflow decrease in baseline amplitude sustaining for at least 10 s. The apnea-hypopnea index (AHI) was calculated as the total of hypopneas and apneas per hour of sleep. The criterion of OSA diagnosis was AHI at least 5 events per hour.

### 2.3. T2D Assessment

The diagnosis of T2D was based on the criteria established by the WHO in 2011 [27]. Participants with fasting blood glucose (FBG) ≥ 7 mmol/L, glycated hemoglobin (HbA1c) ≥ 6.5%, 2 h postload plasma glucose ≥ 11.1 mmol/L, or treatment with antidiabetes medication were regarded as T2D.

### 2.4. Collection and Storage of Plasma Sample

We quickly obtained fasting blood samples after the PSG examination. Procedures for plasma collection, processing, and storage have been previously described [22, 23].

### 2.5. Luminex Assays

The Magnetic Luminex® Assays were magnetic bead-based antibody microarrays that can be used to facilitate the simultaneous quantitation of up to 100 soluble analytes in a single sample [28]. In the study, we custom-made a Luminex panel including CTSS to assess the impact of OSA in T2D (R&D Systems, Inc., Minneapolis, MN, USA). To ensure the accuracy and validity of the results, as previously described, we assessed the Luminex results by the intra-assay variability and standard curve [22]. In this study, the intra-assay coefficient of variation (CV%) of the standard curve was <4.0% (see Table S1 in the Supplementary Materials).

### 2.6. Statistical Analysis

Continuous variables were shown as mean ± standard deviation or median (interquartile range (IQR)) depending on the results of the Kolmogorov–Smirnov test; categorical variables were presented as the number of cases. Normally distributed continuous variables were analyzed by one-way analysis of variance and adjusted by the Bonferroni correction for multiple comparisons. The Kruskal-Wallis test and Bonferroni post hoc analysis were used to evaluate nonnormally distributed continuous variables. The categorical variables were analyzed by the chi-square test with Yates correction. The correlations of continuous variables were analyzed by the Pearson or Spearman correlation coefficient. Multiple linear regression analyses (forced entry method) assessed the influence of variables (age, sex, and other variables with *P* < 0.10 in the univariate model analysis) for plasma CTSS concentration. The receiver operating characteristic (ROC) curves, the area under the curve (AUC), and the sensitivity and specificity of optimal cutoff CTSS plasma concentration were calculated and recorded for the prediction of OSA based on CTSS concentrations. It is statistically significant when the *P* value was less than 0.05. All data were evaluated by SPSS version 25 software (SPSS Inc., Chicago, IL, USA).

## 3. Results and Discussion

### 3.1. Baseline Clinical Characteristics of the Study Population

The demographic and sleep data of the 158 individuals involved in this study are shown in Table 1. Participants were divided into four groups of control, non-OSA&T2D, OSA&non-T2D, and OSA&T2D based on the FBG and AHI values. Participants in the four groups were matched by age, sex, and BMI. Compared with the control group, higher levels of the diastolic blood pressure, ESS score, AHI, percentage of cumulative time with oxygen saturation below 90% (CT90), and arousal index were observed in the OSA&non-T2D group. The “lowest arterial oxygen saturation (SaO_2_)” and “mean SaO_2_” were lower in the OSA&non-T2D group than in the control group. Participants with OSA&T2D had significantly higher diastolic blood pressure, neck circumference, ESS score, AHI, CT90, and arousal index compared to the non-OSA&T2D group. The patients with OSA&T2D had lower “lowest SaO_2_” and “mean SaO_2_” than the non-OSA&T2D patients. There was no significant difference in demographic data between the non-OSA&T2D group and the control group.

Compared to the control group, the levels of total cholesterol, triglyceride, low-density lipoprotein cholesterol, and uric acid were significantly increased in the OSA&non-T2D group (Table 2).

### 3.2. CTSS Levels in the Control Group, the Non-OSA&T2D Group, and the OSA&T2D Group

**Figure 1:**
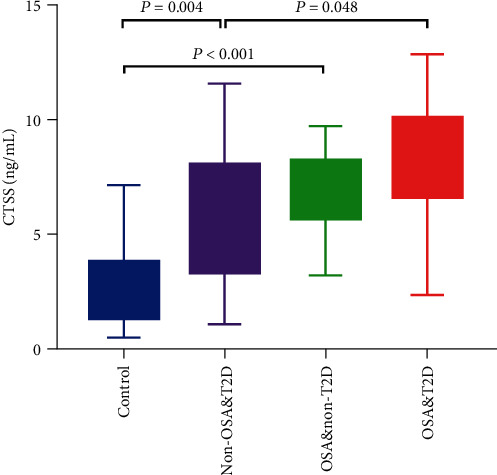
Plasma CTSS levels in the control group, the non-OSA&T2D group, the OSA&non-T2D group, and the OSA&T2D group. Data are median (interquartile range). CTSS: cathepsin S; OSA: obstructive sleep apnea; T2D: type 2 diabetes.

The CTSS levels for the control group, the non-OSA&T2D group, the OSA&non-T2D group, and the OSA&T2D group are shown in Table 2. The CTSS levels were as follows: 1.96 ng/mL (IQR: 1.35-3.78) in the control group, 6.10 ng/mL (IQR: 3.37-8.01) in the non-OSA&T2D group, 6.93 ng/mL (IQR: 5.73-8.18) in the OSA&non-T2D group, and 8.37 ng/mL (IQR: 6.64-10.08) in the OSA&T2D group. The intergroup comparison of CTSS levels was analyzed by the Kruskal-Wallis test, which showed a significant difference. Compared with the control group, both the non-OSA&T2D group and the OSA&non-T2D group showed higher CTSS levels (*P* = 0.004 and *P* < 0.001, respectively). Plasma CTSS concentration was significantly higher in subjects with OSA&T2D than those with non-OSA&T2D (*P* = 0.048). The OSA&T2D group had higher CTSS levels than the OSA&non-T2D group, but there were no statistically significant differences (*P* = 0.160) (Figure 1).

### 3.3. Relationship between CTSS and Clinical Data

We used Spearman's correlation to investigate the correlation between the levels of CTSS and clinical data. Plasma CTSS expression in all individuals was closely related to the AHI (*r* = 0.559, *P* < 0.001), lowest SaO_2_ (*r* = −0.479, *P* < 0.001), mean SaO_2_ (*r* = −0.410, *P* < 0.001), CT90 (*r* = 0.363, *P* < 0.001), and arousal index (*r* = 0.433, *P* < 0.001). Moreover, plasma CTSS expression was significantly related to plasma FBG (*r* = 0.427, *P* < 0.001) and HbA1c (*r* = 0.256, *P* = 0.111) (Table 3).

We used multiple linear regression analyses to evaluate the correlation between CTSS and clinical and biological parameters. As shown in Table 4, with demographics (age, sex, and BMI) and biological parameters (uric acid, alanine aminotransferase (ALT), and *γ*-glutamyl transferase (GGT)) as covariates, it was found that plasma CTSS concentration was positively correlated with the AHI (Beta: 0.386, 95% confidence interval (CI): 21.988 to 57.781; *P* < 0.001), which represents the severity of OSA in this study. In addition, plasma CTSS expression was also positively correlated with the FBG (Beta: 0.340, 95% CI: 328.871 to 921.777; *P* < 0.001).

### 3.4. ROC Curve Analysis of CTSS Levels for the Prediction Value of OSA

**Figure 2:**
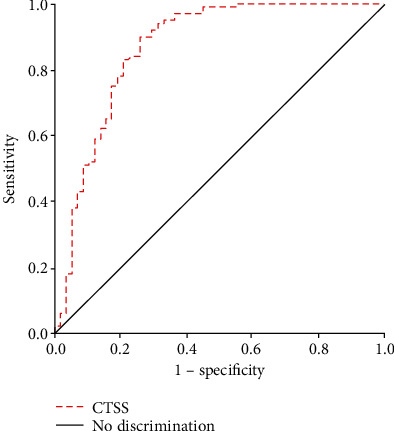
Receiver operating characteristic curves using CTSS levels for prediction of OSA (AUC: 0.868, 95% CI: 0.802-0.934, *P* < 0.001; sensitivity: 90.00%, specificity: 74.10%). CTSS: cathepsin S; OSA: obstructive sleep apnea; AUC: area under the curve; CI: confidence intervals.

We determined based on ROC curves analysis the optimal threshold value for the optimal meeting point between having the greatest sensitivity and specificity for predicting the occurrence of OSA (Figure 2). The optimal cutoff value of plasma CTSS for the diagnosis of OSA was 5.07 ng/mL with a corresponding sensitivity of 90.00% and specificity of 74.10%. The area under the curve was equal to 0.868 (*P* < 0.001), suggesting that CTSS could be a potential indicator of OSA.

### 3.5. Discussion

In this research, we demonstrated that patients with OSA&T2D have significantly elevated plasma CTSS levels, ≈1.5-fold greater than non-OSA&T2D patients. Plasma CTSS expression was positively correlated to the AHI after adjustment of confounding factors. In addition, after adjustment for possible confounders, plasma CTSS levels remained independently interrelated to the FBG. Plasma CTSS was highly discriminatory accurate in predicting OSA. The evidence demonstrated that increased plasma CTSS could be correlated to an inflammatory reaction, OSA severity, and plasma CTSS as a possible prognostic biomarker with a positive diagnostic value on diagnosing OSA.

In addition, our study showed that serum uric acid expression was significantly higher in the OSA&non-T2D group than in the control group, which was in accord with the previous research [29, 30]. Meanwhile, serum uric acid levels were significantly reduced after OSA treatment with continuous positive airway pressure or weight reduction [31]. This finding suggested that it is necessary to diagnose and treat OSA in the early stage.

The relationship between OSA and T2D is a complex interaction. It is established that OSA severity is being increasingly recognized as a possible factor of glucose intolerance, insulin resistance, and diabetes, possibly via the effect of recurrent hypoxemia, inflammation, sympathetic nervous system activation, reactive oxygen species formation, or insulin resistance [4, 5, 32]. Furthermore, it has been hypothesized that diabetes may enhance the predisposition for OSA, due to diabetes-associated autonomic dysfunction influence ventilatory control by abnormalities in autonomic nervous system activity and enhanced central chemoreceptor-mediated gain [6, 7]. Individually, both OSA and T2D are the most common disorders and interrelated to the adverse clinical complications that impose a tremendous burden on public health. In addition, multiple epidemiological studies have indicated a high prevalence of undiagnosed OSA in patients with T2D; screening for sleep patterns and OSA could be of great importance for patients with T2D [10, 33].

The AASM clinical practice guidelines suggest that OSA is diagnosed based on clinical measures, including the subjective assessment of somnolence and the overnight multichannel PSG [26]. Although some screening questionnaires are simple, relatively easy to implement, they are restricted to poor specificity and accuracy for the diagnosis of OSA. Current OSA diagnoses and severity grading are mainly based on PSG, but it is expensive and time-consuming. It is also impractical for the clinical evaluation of large at-risk populations [12]. Therefore, we explore circulating biomarkers, which could provide important information on clinical significance for diagnosing OSA.

CTSS, a cysteine protease, is expressed in endothelial cells and participated in antigen presentation and extracellular matrix remodeling [34]. Previous studies have reported that higher circulating levels of CTSS are associated with increased inflammatory activity and involved in the early dysregulation of glucose and insulin metabolism [16, 35]. Experimental studies suggest that CTSS activity is involved in prediabetic alterations in the liver and in autoimmune diabetes; deficiency in CTSS led to a robust reduction in blood glucose and a decrease in diabetes incidence [17, 18]. Consistent with previous study, we also found an association between elevated plasma levels of CTSS with FBG even after adjustment for age, sex, BMI, AHI, uric acid, ALT, and GGT.

To our knowledge, this study is the first to elucidate the relationship between CTSS and OSA in patients with T2D. However, previous publications have reported associations of CTSS with hypoxia conditions. RNA sequencing analysis showed that CTSS mRNA levels were higher under hypoxic conditions and hypoxia significantly enhanced CTSS activity in aortic endothelial cells [19, 20]. Likewise, in hypoxia-induced rats, CTSS expression progressively upregulated with hypoxia time [36]. These findings were consistent with the related study about human tissue; CTSS upregulated over twofold in the chronic hypoxia muscles of polysaccharide storage myopathy [37]. Although the role of CTSS under OSA-induced CIH conditions was unknown, CTSS played an active role in inflammatory reactions and was upregulated in hypoxic conditions. In our study, the OSA&non-T2D group showed higher CTSS levels compared with the control group. Meanwhile, we also found that plasma CTSS was independently correlated to AHI, even though adjustment was made for age, sex, BMI, FBG, uric acid, ALT, and GGT. An expanded evaluation of CTSS extended to the associations between OSA and T2D, suggesting that inflammatory reactions may be a mechanism by which CTSS contributes to increased OSA risk in patients with T2D. In contrast, neither BMI nor uric acid, ALT, and GGT was found to be associated with CTSS. And intriguingly, the discriminatory accuracy of plasma CTSS was high on diagnosing OSA. Given the high prevalence of undiagnosed OSA in patients with T2D, it was available using plasma CTSS to preliminarily diagnosis OSA in patients with T2D, instead of using complicated overnight multichannel PSG.

To avoid bias in this research, the Luminex experiment was conducted in light of the manufacturer's instructions by the experimenter who does not know the patients' clinical information. Moreover, the confounding effects of risk factors for plasma CTSS levels and AHI were adjusted by multiple linear regression analyses. In addition, plasma CTSS may provide vital value to screen and diagnose OSA in patients with T2D, having important clinical significance. However, the limitations should be noted in our research. First, this study was an observational cross-sectional research, which only showed a correlated relationship. Second, the number of the study population was relatively small; hence, multicenter studies with larger sample sizes were warranted.

## 4. Conclusions

In conclusion, we demonstrated that plasma CTSS expression was significantly elevated in patients with OSA&T2D and was associated with the AHI, which represents the severity of OSA in this study. Plasma CTSS levels provided high discriminatory accuracy values on diagnosing OSA; it might be a potential biomarker with a positive diagnostic value for inflammatory reactions in patients with OSA&T2D. Further, large-scale and prospective studies are explored to verify these findings.

## Figures and Tables

**Table 1:** Baseline clinical characteristics of the study population.

Parameters	Control (*n* = 43)	Non-OSA&T2D (*n* = 15)	OSA&non-T2D (*n* = 66)	OSA&T2D (*n* = 34)	*P* value
Age (years)	50.0 (38.0-64.0)	58.0 (54.0-60.0)	51.0 (46.0-56.5)	54.0 (46.5-57.0)	0.055
Male, *n* (%)	28 (65.1)	10 (66.7)	51 (77.3)	28 (82.4)	0.287
BMI (kg/m^2^)	24.6 (22.2-29.0)	24.3 (21.9-26.4)	26.6 (24.2-28.8)	26.7 (24.0-28.4)	0.095
SBP (mmHg)	120.0 (114.0-134.0)	115.0 (109.0-130.0)	129.0 (117.8-137.3)	128.5 (119.0-139.3)	0.067
DBP (mmHg)	75.0 (69.0-85.0)	70.0 (65.0-78.0)	83.0 (75.0-92.3)^§^	82.0 (77.8-89.0)^††^	<0.001
Current smoker, *n* (%)	11 (25.6)	5 (33.3)	20 (30.3)	17 (50.0)	0.129
Drinker, *n* (%)	5 (11.6)	4 (26.7)	16 (24.2)	11 (32.4)	0.147
LVEF (%)	60.0 (60.0-68.0)	65.0 (62.0-68.0)	67.0 (63.0-70.0)^§§^	64.0 (59.5-68.0)^£^	0.002
Neck circumference (cm)	39.0 (36.0-41.0)	35.0 (33.8-42.0)	41.0 (37.0-43.0)	43.5 (41.3-46.0)^†††££^	<0.001
ESS score	0.0 (0.0-3.0)	6.0 (0.0-9.0)	11.0 (8.0-15.0)^§§§^	11.0 (7.8-15.0)^††^	<0.001
AHI (events/h)	3.1 (2.1-3.9)	3.5 (2.3-7.3)	38.5 (26.2-59.6)^§§§^	47.5 (29.6-73.1)^†††^	<0.001
Lowest SaO_2_ (%)	92.0 (91.0-93.0)	92.0 (88.0-93.0)	80.0 (70.0-86.3)^§§§^	82.0 (68.5-86.3)^†††^	<0.001
Mean SaO_2_ (%)	97.0 (95.8-98.0)	96.0 (95.0-96.0)	94.0 (90.0-95.1)^§§§^	93.5 (92.0-95.0)^††^	<0.001
CT90 (%)	0.0 (0.0-0.0)	0.0 (0.0-0.2)	6.4 (0.5-16.8)^§§§^	4.9 (0.2-20.6)^††^	<0.001
Arousal index (events/h)	0.7 (0.0-3.2)	3.5 (0.3-6.7)	36.7 (12.3-58.1)^§§§^	11.9 (7.2-16.7)^†^	<0.001

Data are presented as median (interquartile range) or *n* (%), unless otherwise stated. ^§§§^*P* < 0.001, ^§§^*P* < 0.01, and ^§^*P* < 0.05 between OSA&non-T2D and control. ^†††^*P* < 0.001, ^††^*P* < 0.01, and ^†^*P* < 0.05 between OSA&T2D and non-OSA&T2D. ^£££^*P* < 0.001, ^££^*P* < 0.01, and ^£^*P* < 0.05 between OSA&T2D and OSA&non-T2D. OSA: obstructive sleep apnea; T2D: type 2 diabetes; BMI: body mass index; SBP: systolic blood pressure; DBP: diastolic blood pressure; LVEF: left ventricular ejection fraction; ESS: Epworth Sleepiness Scale; AHI: apnea-hypopnea index; SaO_2_: arterial oxygen saturation; CT90: percentage of cumulative time with oxygen saturation below 90%.

**Table 2:** Biological parameters and CTSS plasma levels of the study population.

Parameters	Control (*n* = 43)	Non-OSA&T2D (*n* = 15)	OSA&non-T2D (*n* = 66)	OSA&T2D (*n* = 34)	*P* value
FBG (mmol/L)	5.1 (4.9-5.3)	6.1 (4.9-6.7)^∗^	5.2 (5.0-5.6)	7.4 (6.0-9.5)^£££^	<0.001
HbA1c (%)	5.6 (5.4-6.0)	6.6 (6.1-7.3)	5.7 (5.3-5.9)	7.1 (6.5-8.5) ^£^	<0.001
GPS (%)	13.7 (12.6-14.5)	17.4 (15.3-21.4)^∗∗^	12.8 (11.9-13.6)	17.6 (15.0-21.3)^£££^	<0.001
Duration of T2D (years)	/	24.0 (14.0-72.0)	/	36.0 (18.0-88.0)	0.462
Total cholesterol (mmol/L)	4.6 (4.1-5.2)	4.1 (3.5-4.9)	5.2 (4.6-6.0)^§§^	4.6 (3.8-5.7)^£^	<0.001
Triglyceride (mmol/L)	1.1 (0.8-1.8)	1.3 (0.9-1.4)	1.6 (1.2-2.2)^§^	1.7 (1.3-2.1)	0.001
HDL-C (mmol/L)	1.2 (1.1-1.4)	1.2 (1.0-1.6)	1.2 (1.0-1.3)	1.1 (0.9-1.3)	0.231
LDL-C (mmol/L)	2.7 (2.3-3.2)	2.4 (1.7-3.3)	3.3 (2.9-3.9)^§§^	2.7 (2.1-3.5)^£^	<0.001
hs-CRP (mg/L)	0.8 (0.3-2.1)	2.0 (0.6-7.0)	1.3 (0.5-2.3)	1.7 (1.0-4.7)	0.018
Platelet (G/L)	216.5 (176.3-262.8)	228.0 (171.0-280.0)	226.0 (198.0-257.0)	212.0 (188.5-260.5)	0.777
Leukocyte (G/L)	5.6 (4.8-7.1)	6.3 (5.2-7.9)	6.1 (5.3-7.3)	6.9 (5.5-7.7)	0.289
Uric acid (*μ*mol/L)	343.4 (294.5-397.3)	337.6 (257.4-386.4)	399.0 (330.8-484.3)^§^	393.9 (342.2-472.7)	0.001
ALT (U/L)	20.5 (12.8-31.3)	25.0 (20.0-41.0)	26.0 (16.0-36.5)	29.0 (16.5-43.5)	0.082
GGT (U/L)	23.0 (16.0-35.0)	28.5 (21.5-48.5)	31.5 (20.0-48.5)	41.5 (27.0-63.0)	0.004
Homocysteine (*μ*mol/L)	10.2 (8.5-15.4)	12.7 (8.9-15.7)	12.0 (9.3-15.7)	10.7 (8.8-16.3)	0.857
Urea nitrogen (mmol/L)	4.7 (4.1-5.9)	5.9 (4.2-7.8)	5.0 (4.5-5.9)	5.0 (4.2-6.3)	0.354
Creatinine (*μ*mol/L)	65.9 (54.1-74.8)	59.7 (58.4-64.2)	70.7 (60.8-78.9)	64.1 (57.3-76.4)	0.027
ALP (U/L)	73.0 (63.0-90.0)	72.0 (60.0-91.8)	72.0 (61.5-84.5)	78.0 (62.0-88.5)	0.919
AST (U/L)	20.0 (18.0-24.3)	21.0 (16.0-35.0)	22.0 (19.0-26.0)	22.0 (18.5-31.5)	0.522
Creatine kinase (U/L)	96.0 (70.0-121.0)	66.0 (54.0-85.5)	110.0 (70.0-145.0)	94.0 (71.0-128.5)	0.071
CTSS (ng/mL)	1.96 (1.35-3.78)	6.10 (3.37-8.01)^∗∗^	6.93 (5.73-8.18)^§§§^	8.37 (6.64-10.08)^†^	<0.001

Data are presented as median (interquartile range). ^∗∗∗^*P* < 0.001, ^∗∗^*P* < 0.01, and ^∗^*P* < 0.05 between non-OSA&T2D and control. ^§§§^*P* < 0.001, ^§§^*P* < 0.01, and ^§^*P* < 0.05 between OSA&non-T2D and control. ^†††^*P* < 0.001, ^††^*P* < 0.01, and ^†^*P* < 0.05 between OSA&T2D and non-OSA&T2D. ^£££^*P* < 0.001, ^££^*P* < 0.01, and ^£^*P* < 0.05 between OSA&T2D and OSA&non-T2D. OSA: obstructive sleep apnea; T2D: type 2 diabetes; FBG: fasting blood glucose; HbA1c: glycated hemoglobin; GPS: glycosylated serum protein; HDL-C: high-density lipoprotein cholesterol; LDL-C: low-density lipoprotein cholesterol; hs-CRP: high-sensitivity C-reactive protein; ALT: alanine aminotransferase; GGT: *γ*-glutamyl transferase; ALP: alkaline phosphatase; AST: aspartate aminotransferase; CTSS: cathepsin S.

**Table 3:** Correlation between CTSS and clinical and laboratory variables.

Parameters	Rho	*P* value
AHI	0.559	<0.001
Lowest SaO_2_	-0.479	<0.001
Mean SaO_2_	-0.410	<0.001
CT90	0.363	<0.001
Arousal index	0.433	<0.001
FBG	0.427	<0.001
HbA1c	0.256	0.111

Spearman's rho was used for correlations between CTSS and clinical and laboratory parameters. CTSS: cathepsin S; AHI: apnea-hypopnea index; SaO_2_: arterial oxygen saturation; CT90: percentage of cumulative time with oxygen saturation below 90%; FBG: fasting blood glucose; HbA1c: glycated hemoglobin.

**Table 4:** Association between levels of CTSS and variables in univariate and multiple linear regression models.

	Univariate models			Multiple model (*R*^2^ = 0.373, *P* < 0.001)		
	Beta	95% CI	*P* value	Beta	95% CI	*P* value
Age (years)	-0.005	-51.183 to 48.343	0.955	0.034	-33.850 to 54.399	0.645
Sex (female vs. male)	0.186	124.692 to 2497.537	0.031	0.066	-603.815 to 1524.928	0.393
BMI (kg/m^2^)	0.116	-34.536 to 227.965	0.148	-0.054	-189.882 to 89.546	0.478
LVEF (%)	0.022	-69.687 to 91.954	0.786			
AHI (events/h)	0.506	39.757 to 69.073	<0.001	0.386	21.988 to 57.781	<0.001
FBG (mmol/L)	0.444	602.300 to 1165.896	<0.001	0.340	328.871 to 921.777	<0.001
Total cholesterol (mmol/L)	0.075	-194.509 to 541.078	0.353			
Triglyceride (mmol/L)	0.085	-158.839 to 527.189	0.291			
LDL-C (mmol/L)	0.095	-204.688 to 815.319	0.239			
hs-CRP (mg/L)	0.074	-59.811 to 161.904	0.364			
Uric acid (*μ*mol/L)	0.136	-0.594 to 8.440	0.088	-0.095	-7.434 to 1.876	0.239
ALT (U/L)	0.171	1.374 to 40.573	0.036	0.012	-17.892 to 20.757	0.883
GGT (U/L)	0.208	4.290 to 29.517	0.009	0.113	-3.932 to 21.711	0.172
Creatinine (*μ*mol/L)	0.087	-9.832 to 33.883	0.279			
Creatine kinase (U/L)	0.013	-3.387 to 3.966	0.877			

The multiple linear regression model includes age, sex, BMI, and other variables with *P* < 0.10 in univariate model analysis. *R*^2^: adjusted *R*^2^ of the multiple linear regression model; CI: confidence intervals; CTSS: cathepsin S; BMI: body mass index; LVEF: left ventricular ejection fraction; AHI: apnea-hypopnea index; FBG: fasting blood glucose; LDL-C: low-density lipoprotein cholesterol; hs-CRP: high-sensitivity C-reactive protein; ALT: alanine aminotransferase; GGT: *γ*-glutamyl transferase.

## Data Availability

The datasets used and analyzed during the current study are available from the corresponding author on reasonable request.

## Supplementary Materials

Supplementary Materials Table S1: evaluation of Luminex assay standard curves and intra-assay variability for CTSS levels determined using the Luminex.
