# Supplementary material for: Usefulness of Cathepsin S to Predict Risk for Obstructive Sleep Apnea among Patients with Type 2 Diabetes
Source: Dis Markers. 2020 Sep 25;2020:8819134. doi: 10.1155/2020/8819134 (PMC7533779; doi:10.1155/2020/8819134)
Supplement: Supplementary Materials — Table S1: evaluation of Luminex assay standard curves and intra-assay variability for CTSS levels determined using the Luminex. [file 8819134.f1.docx]

Table S1: Evaluation of Luminex assay standard curves and intra-assay variability for CTSS levels determined using the Luminex

| Cytokines | LoB(pg/L) | LoD(pg/L) | LLoQ(pg/L) | ULoQ(pg/L) | Intra-assay (CV%) |
| --- | --- | --- | --- | --- | --- |
| CTSS | 28.7 | 30.9 | 98.8 | 22570 | 0.26-3.73 |

LoB: Limit of Blank; LoD: Limit of Detection; LLoQ: Lower Limit of Quantitation; ULoQ: Upper Limit of Quantitation; CV: Coefficient of variation; CTSS: Cathepsin S
